# Clubhouse Model of Psychiatric Rehabilitation in China to Promote Recovery of People With Schizophrenia: A Systematic Review and Meta-Analysis

**DOI:** 10.3389/fpsyt.2021.730552

**Published:** 2021-09-13

**Authors:** Haohao Yan, Yudan Ding, Wenbin Guo

**Affiliations:** ^1^National Clinical Research Center for Mental Disorders, and Department of Psychiatry, The Second Xiangya Hospital of Central South University, Changsha, China; ^2^Department of Psychiatry, The Third People's Hospital of Foshan, Foshan, China

**Keywords:** clubhouse, psychiatric rehabilitation, china, schizophrenia, meta-analysis

## Abstract

**Background:** Whether the clubhouse model of psychiatric rehabilitation is well-implemented in China and whether patients with schizophrenia successfully achieve symptom remission and functional recovery through engaging in the clubhouse remain unclear.

**Methods:** Seven electronic databases were searched for relevant articles from inception to April 21, 2021. Quality assessment, data synthesis, and subgroup analysis were performed on the included studies.

**Results:** Seven randomized controlled studies with 682 participants were included in the present meta-analysis. The clubhouse model of psychiatric rehabilitation has a significant effect on promoting the remission of psychiatric symptoms, especially negative symptoms. However, it does not show a definite effect on promoting recovery of positive symptoms. The clubhouse model of psychiatric rehabilitation has a significant effect on promoting social functioning recovery, reducing the family burden, improving the quality of life, and promoting the remission of depressive and anxiety symptoms of patients with schizophrenia in China.

**Conclusions:** Our findings suggest that the clubhouse model of psychiatric rehabilitation can promote the remission of symptoms and functional recovery of Chinese with schizophrenia. It may be suitable to address the urgent need for better mental health services in China.

## Introduction

Although China has made substantial advancements in the treatment and management of mental disorders, these disorders still pose a heavy burden. An epidemiological survey estimated that about 173 million Chinese suffer from diagnosable mental disorders, of whom 158 million have never received any treatment (1). Furthermore, ~16 million Chinese are affected by severe mental illness (SMI) (1). A recent study estimated that the lifetime prevalence of most mental disorders and schizophrenia in China was 1.3% (~17 million Chinese citizens) and 0.6% (~8 million Chinese citizens), respectively (2). Globally, the economic burden of schizophrenia was estimated to range from 0.02 to 1.65% of the gross domestic product (3). In high-income countries, SMI has already become the leading cause of disability. Other countries including China may experience a similar reality (4). Despite substantial advances in clinical treatment, patients with SMI continue to suffer poor social predicaments, including high rates of unemployment, stigma, and homelessness (5–7). These challenges highlight the necessity of delivering mental health service that addresses both social and clinical needs. A primary difficulty in developing mental health service has been that professionals and patients with SMI often disagree on treatment goals. Professionals often emphasize multiple medications to relieve symptoms of a biological disorder, whereas patients with SMI emphasize the need for supports to promote functional recovery and to reduce psychological distress. Similarly, professionals highly value symptom control as a primary goal, whereas patients with SMI prioritize having a satisfying and meaningful life (8). In part because of the difference, some patients with mental disorders avoid the mental health system. In response to patients' dissatisfaction with the traditional emphasis on symptom control and stability, mental health policy makers in some countries now advocate the concept of personal recovery (9).

Despite substantial advancements in pharmacological treatment of patients with SMI, medications alone are not sufficient to achieve a complete functional recovery and symptom remission (10). In the recent years, treatment of SMI has gradually shifted from stabilization and management of clinical symptoms to the more ambitious and much broader goal of achieving functional recovery. In this process, psychiatric rehabilitation (also known as psychosocial rehabilitation) has been accepted by the mental health field as one of the preferred methods for helping patients with mental disorders (11). Psychiatric rehabilitation aims to promote recovery and community integration and to improve the quality of life of patients with mental disorders using the development of skills and supports as its primary types of interventions. The term “psychiatric rehabilitation” reflects the focus of the mental health field on patients with psychiatric disabilities and their improved abilities within their specific preferred role in the “real” world (12, 13). Although psychosocial rehabilitation services focus on achieving personal recovery, they are still hard to access for patients with SMI in the developed countries such as America, let alone the developing countries including China. In America, <5% of patients with SMI can access high-quality psychiatric rehabilitation services (11). In China, 1.3% of patients with SMI can access psychiatric rehabilitation services (14). Several psychiatric rehabilitation models, such as the clubhouse model, workstation programs, farming programs, and family-based collaborative care model, have emerged in China (15). The clubhouse model of psychiatric rehabilitation has been in existence for over 70 years around the world and helps thousands of people with SMI (16). Clubhouses define their service users as members, rather than clients or patients, because all members actively engage in all aspects of the clubhouse compared with other passive service users. This means that clubhouse members are afforded self-determination. Clubhouses provide social events, work experiences, and housing to strengthen and increase the social networks of people with SMI. One of the outstanding characteristics of the clubhouse is that it creates an environment through peer support to promote a sense of community and belonging (17–19). The clubhouse model of psychiatric rehabilitation is a promising practice (19–21). In mainland China, the first accredited clubhouse, the Changsha Heart Wing Clubhouse, was founded in 2007. At present, six clubhouses are operating in mainland China (22). However, some concerns aroused during the glocalization of clubhouse models, including whether and how it is affected by different societal contexts. Some studies indicated that the clubhouse, to some extent, modifies or translates the model to fit its national and local context (23, 24). China is characterized by Confucian social ethics, which means that Chinese rely more on the family and organized civil society. The family is regarded as the main caregivers for people with mental disorders. Moreover, significant differences exist in the welfare system, politics, economy, and culture between China and other countries. Whether the clubhouse model of psychiatric rehabilitation is well-implemented in China and whether patients with schizophrenia successfully achieve symptom remission and functional recovery through engaging in the clubhouse remain unclear. To address these concerns, we conducted the present systematic review and meta-analysis.

## Methods

A systematic review and meta-analysis was performed in accordance with the Preferred Reporting Items for Systematic Reviews and Meta-Analyses (PRISMA) guidelines (25). The review protocol was registered at PROSPERO as CRD 42021251534.

### Search Strategy

Two authors (YD and HY) independently identified relevant articles published in Scopus, Embase, PubMed, Web of Science, China National Knowledge Infrastructure, WANFANG DATA, and VIP Database for Chinese Technical Periodicals from inception to April 21, 2021. We applied the following terms in retrieving the PubMed database: (China OR Chinese) AND (psychiatric rehabilitation OR psychosocial rehabilitation OR clubhouse OR clubhouse model). Some modifications were made as required for retrieving other databases. In addition, the reference lists of the included articles were hand-searched to find additional relevant articles.

### Study Selection Criteria

Longitudinal, controlled studies that aimed to determine whether the clubhouse model of psychiatric rehabilitation in China can promote recovery of people with schizophrenia were included in the review. In order to ensure the credibility of the conclusions, we only included studies which reported that the clubhouses had been accredited by Clubhouse International and/or reported following the International Clubhouse Standards at the time of the study. Individual and cluster randomized controlled trials were included in the meta-analysis. Longitudinal, case-controlled studies were included in the qualitative synthesis. Self-controlled case series studies and conference abstracts were excluded.

### Data Extraction and Quality Assessment

Two authors (YD and HY) independently extracted the following data from the included articles: the name of the first author; publication dates of the included articles; the cities or regions; the name of the clubhouse; the type of study design; participants; the guideline used in diagnosis; the interventions of the experimental and control groups; the time of intervention; dropout data; the sample sizes of the experimental and control groups; the sex ratio, illness duration, educational level, and marital status of the experimental and control groups; the scales used for assessment; the post-treatment mean score and standard deviation of the experimental and control groups; employment data; and relapse data.

The Cochrane Collaboration risk of bias tool was applied to assess the quality of studies included in the meta-analysis. A rating of low, high, or unclear risk of bias was given for the following domains: sequence generation, allocation concealment, masking of assessors, selective outcome reporting, incomplete data, and other sources of bias. Blinding of participants and workers delivering the intervention was not possible to implement due to the nature of the interventions. Therefore, this criterion was excluded in the quality assessment. The Mixed Methods Appraisal Tool was applied to assess the risk of bias of all non-randomized controlled studies (26, 27).

In data extraction and quality assessment, a third team member (WG) performed the verification. All discrepancies were discussed and resolved by the three authors.

### Data Analysis

Data analyses were performed using RevMan 5.4 software. For continuous variables, the standardized mean difference (SMD) with 95% confidence interval (CI) was calculated using a random effect model in order to pool outcomes of different scales. SMD is a summary statistic that represents the size of the intervention effect in a study relative to the variability (clubhouse model of psychiatric rehabilitation) observed in that study. The following cutoffs were used to guide the interpretation of the strength of effect: 0.2–0.5 represents a “small” effect, 0.5–0.8 represents a “medium” effect, and more than 0.8 represents a “large” effect (28). For any scales in which a higher score means better outcome, the mean scores were inverted before calculating the SMD. For binary variables, the odds ratio and 95% CI were calculated using a random effect model. Chi-square statistic (significance level of *p* < 0.05) and *I*^2^ (significance level of *I*^2^ > 50%) were applied to assess heterogeneity across studies. Subgroup analysis was performed according to participants, scales applied for assessment, and time points to explore the potential source of heterogeneity.

## Results

### Literature Search

**Figure 1:**
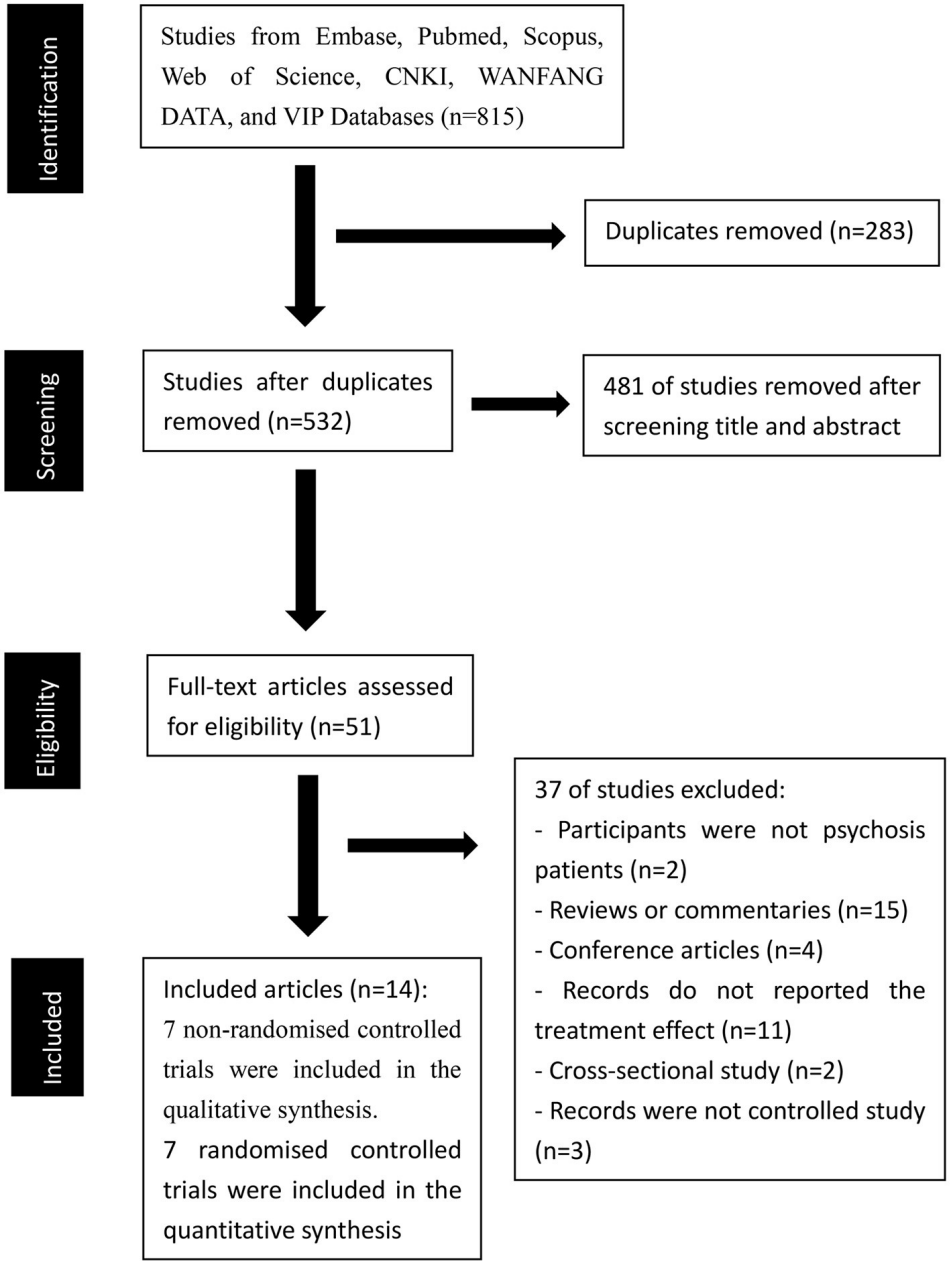
PRISMA diagram of the article selection.

Our initial search identified 815 records in the seven electronic databases (88 records in Scopus, 192 records in Embase, 115 records in PubMed, 71 records in Web of Science, 139 records in China National Knowledge Infrastructure, 180 records in WANFANG DATA, and 30 records in VIP Database for Chinese Technical Periodicals). A total of 764 records were excluded after removing duplicates (283 articles) and screening titles and abstracts (481 articles). Accordingly, 51 potentially relevant articles were retrieved for detailed full-text evaluation. We excluded 37 records (participants in 2 records were not psychosis patients; 15 records were reviews or commentaries; 4 records were conference articles; 11 records did not report the treatment effect of the clubhouse model on psychiatric rehabilitation; 2 records were cross-sectional studies, 3 records were not controlled studies). Finally, 14 articles were included [7 non-randomized controlled trials (29–35) were included in the qualitative synthesis, and 7 randomized controlled trials (36–42) were included in the quantitative synthesis]. A PRISMA diagram of the article selection is shown in Figure 1.

### Characteristics of Included Studies

Seven randomized controlled studies (36–42) were included in this meta-analysis. All included studies had a total of 682 participants (286 males and 396 females) with a median sample size of 90, ranging from 51 to 160. In four studies (38, 39, 41, 42), the participants were patients with first-episode schizophrenia. In three other studies (36, 37, 40), the participants were patients with schizophrenia and were not specific to first-episode schizophrenia. In the study of Xiang et al. (37), the participants were women with schizophrenia. Four studies (37–39, 41) applied the third version of the Chinese Classification of Mental Disorders (CCMD-3), 2 studies (40, 42) applied the 10th version of the International Statistical Classification of Diseases and Related Health Problems (ICD-10), and 1 study (36) applied the 4th version of the Diagnostic and Statistical Manual of Mental Disorders (DSM-IV) guidelines for diagnosis. In all seven included studies, the participants were in a stable state with a low-dose medication treatment. In five studies (37–39, 41, 42), the clubhouse model of psychiatric rehabilitation plus general psychiatric rehabilitation was applied in the experimental group, while only general psychiatric rehabilitation was applied in the control group. In one study (40), the clubhouse model of psychiatric rehabilitation was applied in the experimental group, and psychiatric rehabilitation was not applied in the control group. In another study (36), the clubhouse model of psychiatric rehabilitation was applied in the experimental group, while general psychiatric rehabilitation was applied in the control group. The interventions in general psychiatric rehabilitation included holding lectures about keeping healthy and medication use management and giving advices about diet and excise. The time of intervention was 18 months in 1 study (37), 12 months in 2 studies (41, 42), 6 months in 3 studies (36, 38, 39), and 3 months in 1 study (40). Four studies measured the outcomes at different time points (37–39, 41). Five studies reported the dropout data (36–38, 40, 42). Positive and Negative Syndrome Scale (PANSS) and Brief Psychiatric Rating Scale were applied to assess the severity of psychiatric symptoms. The Activity of Daily Living Scale, Personal and Social Performance Scale, and Social Disability Screening Schedule were applied to assess social functioning. The Family Burden Scale (FBS) was applied to assess family burden. The Schizophrenia Quality of Life Scale and life satisfaction index (LSI) were applied to assess the quality of life. The Self-Rating Depression Scale and Hamilton Depression Scale were applied to assess depressive symptoms. The Self-Rating Anxiety Scale and Hamilton Anxiety Scale were applied to assess anxiety symptoms. Only one study (36) reported employment and relapse data. The characteristics of the seven included articles are summarized in Table 1.

**Table 1:** Characteristics of the included randomized controlled studies.

**References**	**City**	**Participants**	**Diagnosis**	**Intervention**	**The time of intervention**	**Experimental group: sample size and demographic characteristic**	**Control group: sample size and demographic characteristic**	**Dropout data**
Xiang et al., (37)	Wuhan	Women with schizophrenia	CCMD-3	Experimental group: A+B Control group: B	18 months	45 (Man: 0; Age: 36.31 ± 4.79 (y); Marital status: Single 30, Married 15; Educational level: Primary and Secondary 10, Tertiary 35)	45 (Man: 0; Age: 37.42 ± 5.33 (y); Marital status: Single 27, Married 18; Educational level: Primary and Secondary 8, Tertiary 37)	clubhouse 0; control 0
Shen et al. (41)	Shenzhen	Patients with first-episode schizophrenia	CCMD-3	Experimental group: A+B Control group: B	12 months	81 [Man:38; Age: 45.26 ± 8.41 (y)]	30 [Man:13; Age: 45.18 ± 8.61 (y)]	NA
Liu et al. (39)	Wuhan	Patients with first-episode schizophrenia	CCMD-3	Experimental group: A+B Control group: B	6 months	58 [Man: 25; Age 37.34 ± 4.12 (y); Educational level: Primary 8, Secondary 38, Tertiary 12]	40 [Man: 17; Age 38.72 ± 4.62 (y); Educational level: Primary 6, Secondary 26, Tertiary 8]	NA
Liu et al. (38)	Guangzhou	Patients with first-episode schizophrenia	CCMD-3	Experimental group: A+B Control group: B	6 months	45 [Man: 20; Age: 40.50 ± 2.30 (y)]	45 [Man: 18; Age 38.70 ± 3.90 (y)]	clubhouse 0; control 0
Huang et al. (2)	Chongqin	Patients with first-episode schizophrenia	ICD-10	Experimental group: A+B Control group: B	12 months	80 [Man: 42; Age: 40.82 ± 8.96 (y); illness duration:1.37 ± 0.28 (y); Educational level: Primary 0, Secondary 55, Tertiary 25]	80 [Man: 41; Age: 40.17 ± 8.05 (y); illness duration: 1.19 ± 0.22 (y); Educational level: Primary 0, Secondary 56, Tertiary 24]	clubhouse 0; control 0
Chen et al., (36)	Chengdu	Patients with schizophrenia	DSM-IV	Experimental group: A Control group: B	6 months	28 [Man: 20; Age 38.96 ± 10.23 (y); Illness duration: 16.97 ± 8.83 (y); Educational level: Primary 2, Secondary 22, Tertiary 4; Marital status: Single 12, Married 5, Separated 11]	23 [Man: 14; Age: 39.13 ± 11.57 (y); Illness duration: 17.30 ± 10.19 (y); Educational level: Primary 1, Secondary 17, Tertiary 5; Marital status: Single 10, Married 6, Separated 7[	clubhouse 0; control 5
Yang et al. (40)	Chongqin	Patients with schizophrenia	ICD-10	Experimental group: A Control group: None	3 months	41 [Man: 18; Age:43 ±10 (y); Illness duration: 7–30 (y); Educational level: Primary 4, Secondary 28, Tertiary 9]	41 [Man: 20; Age: 44 ± 10 (y); Illness duration 8–27 (y); Educational level: Primary 5, Secondary 31, Tertiary 5]	clubhouse 2; control 2

*CCMD-3, the third version of Chinese Classification of Mental Disorders; ICD-10, the 10th version of the International Statistical Classification of Diseases and Related Health Problems; DSM-IV, the 4th version of the Diagnostic and Statistical Manual of Mental Disorders; A, clubhouse model of psychiatric rehabilitation; B, general psychiatric rehabilitation; NA, not available*.

The results of quality assessment are shown in Supplementary Figure 1. Overall, the included studies had low to moderate risk of bias. No studies adequately described the allocation concealment. Only one study (42) clearly described the blinding of outcomes assessment.

The characteristics and quality assessment of the non-randomized controlled trials are summarized in Supplementary Tables 1, 2, respectively.

### Meta-Analytical Results

#### Psychiatric Symptoms

**Figure 2:**
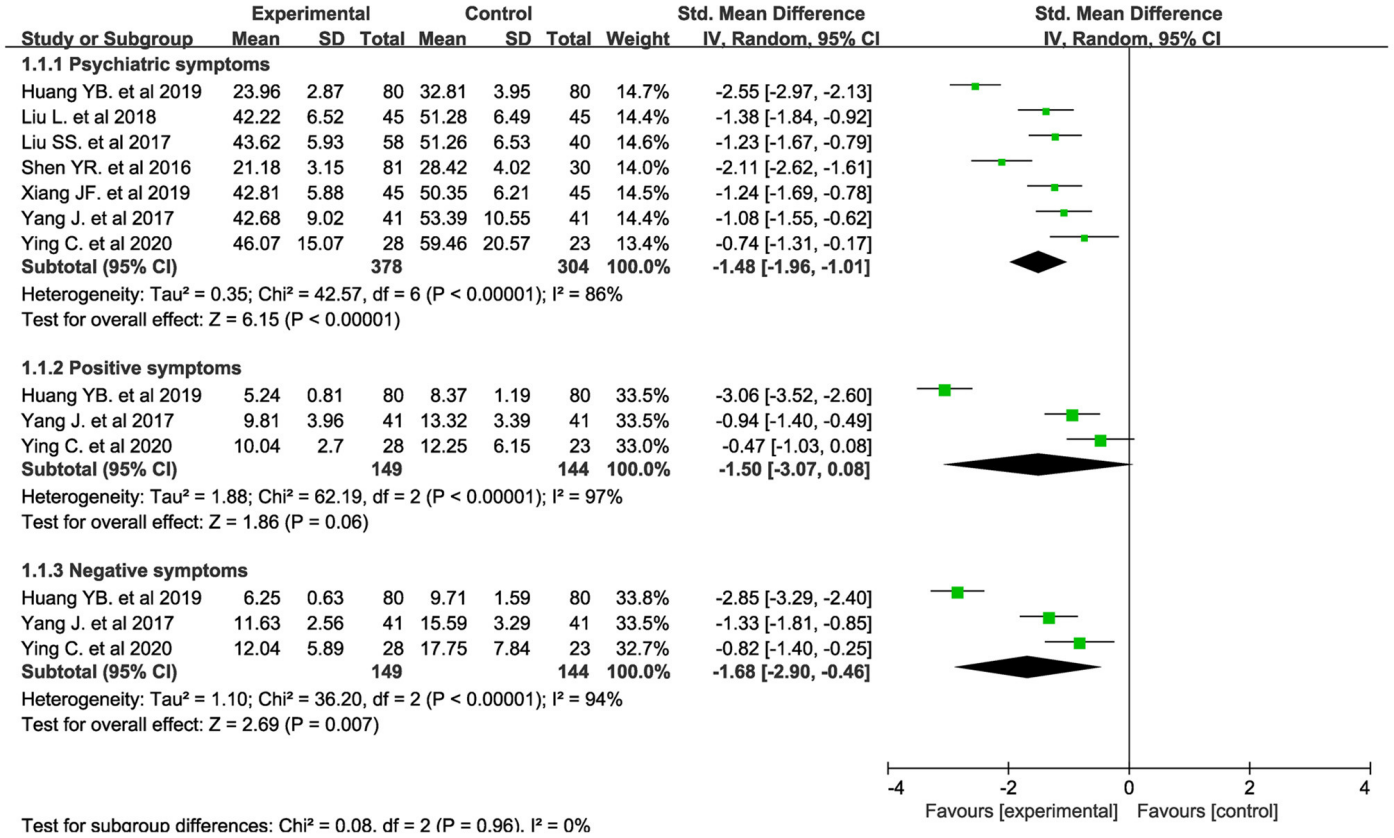
Forest plot about the treatment effectiveness of the clubhouse model of psychiatric rehabilitation on psychiatric symptoms, negative symptoms, and positive symptoms.

The clubhouse model of psychiatric rehabilitation had a significant effect on promoting the remission of psychiatric symptoms (SMD = −1.48, *p* < 0.001, 95% CI = −1.96 to −1.01, *I*^2^ = 86%, k = 7, *n* = 682, Figure 2) (36–42). The pooled SMD indicated that the clubhouse model of psychiatric rehabilitation had a strong effect on promoting the remission of psychiatric symptoms when the outcomes measured with PANSS were pooled (SMD = −1.38, *p* < 0.001, 95% CI = −1.89 to −0.87, *I*^2^ = 86%, k = 6, *n* = 571, Supplementary Figure 2) (36–40, 42). The clubhouse model of psychiatric rehabilitation had a strong effect on promoting the remission of psychiatric symptoms of patients with first-episode schizophrenia (SMD = −1.49, *p* < 0.001, 95% CI = −2.25 to −0.72, *I*^2^ = 91%, k = 4, *n* = 399, Supplementary Figure 3) (36, 38, 39, 42). We also pooled the outcomes measured at different time points. The clubhouse model of psychiatric rehabilitation had a medium effect on promoting the remission of psychiatric symptoms when it was performed for 3 months (SMD = −0.67, *p* < 0.001, 95% CI = −0.94 to −0.41, *I*^2^ = 33%, k = 4, *n* = 381, Supplementary Figure 4) (38–41). The clubhouse model of psychiatric rehabilitation had a strong effect on promoting the remission of psychiatric symptoms when it was performed for 6 months (SMD = −1.18, *p* < 0.001, 95% CI = −1.42 to −0.94, *I*^2^ = 1%, k = 4, *n* = 350, Supplementary Figure 4) (36, 38, 39, 41) and 12 months (SMD = −1.71, *p* = 0.01, 95% CI = −3.02 to −0.40, *I*^2^ = 96%, k = 3, *n* = 361, Supplementary Figure 4) (37, 41, 42).

The clubhouse model of psychiatric rehabilitation had a strong effect on promoting recovery of negative symptoms (SMD = −1.68, *p* = 0.007, 95% CI = −2.90 to −0.46, *I*^2^ = 94%, k = 3, *n* = 293, Figure 2) (36, 40, 42). However, it did not show a definite effect on promoting recovery of positive symptoms (SMD = −1.50, *p* = 0.06, 95% CI = −3.07 to 0.08, *I*^2^ = 97%, k = 3, *n* = 293, Figure 2) (36, 40, 42).

#### Social Functioning

**Figure 3:**
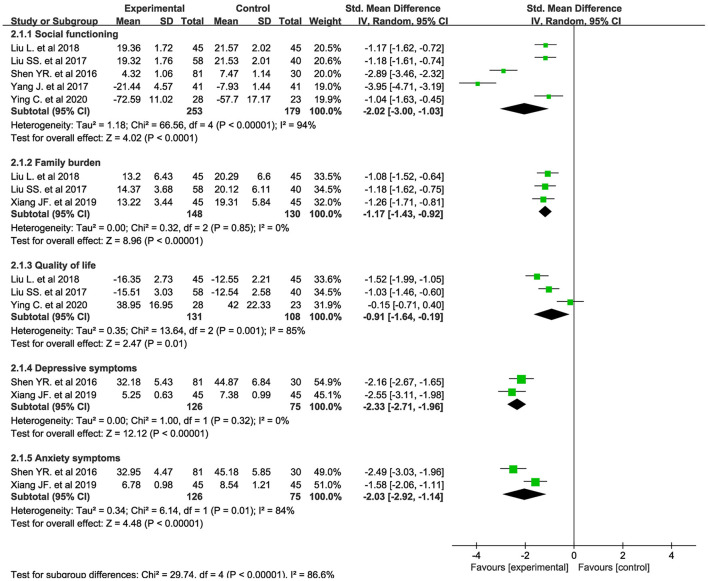
Forest plot about the treatment effectiveness of the clubhouse model of psychiatric rehabilitation on social functioning, family burden, quality of life, and depressive and anxiety symptoms.

The clubhouse model of psychiatric rehabilitation had a strong effect on promoting social functioning recovery (SMD = −2.02, *p* < 0.001, 95% CI = −3.00 to −1.03, *I*^2^ = 94%, k = 5, *n* = 432, Figure 3) (36, 38–41). In addition, it had a strong effect on promoting social functioning recovery in patients with first-episode schizophrenia (SMD = −1.73, *p* = 0.001, 95% CI = −2.75 to −0.71, *I*^2^ = 93%, k = 3, *n* = 299, Supplementary Figure 5) (38, 39, 41). We pooled the outcomes measured at different time points. The clubhouse model of psychiatric rehabilitation had a strong effect on promoting social functioning recovery when it was performed for 3 months (SMD = −1.48, *p* = 0.009, 95% CI = −2.59 to −0.37, *I*^2^ = 95%, k = 4, *n* = 381, Supplementary Figure 6) (38–41) and 6 months (SMD = −1.36, *p* < 0.001, 95% CI = −1.80 to −0.92, *I*^2^ = 69%, k = 4, *n* = 350, Supplementary Figure 6) (36, 38, 39, 41). One study reported the promotion of social functioning recovery when this intervention was performed for 12 months (SMD = −2.89, *p* < 0.001, 95% CI = −3.46 to −2.32, *n* = 111, Supplementary Figure 6) (41).

#### Family Burden

Family burden was assessed using the FBS. The clubhouse model of psychiatric rehabilitation had a strong effect on reducing the family burden (SMD = −1.17, *p* < 0.001, 95% CI = −1.43 to −0.92, *I*^2^ = 0%, k = 3, *n* = 278, Figure 3) (37–39). In addition, it had a strong effect on reducing the family burden of patients with first-episode schizophrenia (SMD = −1.13, *p* < 0.001, 95% CI = −1.44 to −0.82, *I*^2^ = 0%, k = 2, *n* = 188, Supplementary Figure 7) (38, 39). The clubhouse model of psychiatric rehabilitation had a medium effect on reducing the family burden when it was performed for 3 months (SMD = −0.77, *p* ≤ 0.001, 95% CI = −1.07 to −0.47, *I*^2^ = 0%, k = 2, *n* = 188, Supplementary Figure 8) (38, 39) and a strong effect when it was performed for 6 months (SMD = −1.13, *p* < 0.001, 95% CI = −1.44 to −0.82, *I*^2^ = 0%, k = 2, *n* = 188, Supplementary Figure 8) (38, 39). One study reported the reduction of family burden when this intervention was performed for 12 months (SMD = −0.85, *p* < 0.001, 95% CI = −1.29 to −0.42, *n* = 90, Supplementary Figure 8) (37). Another study reported the reduction of family burden when this intervention was performed for 18 months (SMD = −1.26, *p* < 0.001, 95% CI = −1.71 to −0.81, *n* = 90, Supplementary Figure 8) (37).

#### Quality of Life

The clubhouse model of psychiatric rehabilitation had a strong effect on improving the quality of life of patients with schizophrenia (SMD = −0.91, *p* = 0.01, 95% CI = −1.64 to −0.19, *I*^2^ = 85%, k = 3, *n* = 239, Figure 3) (36, 38, 39). Two studies (38, 39) measured the treatment effect on improving the quality of life of patients with first-episode schizophrenia using LSI scales. The clubhouse model of psychiatric rehabilitation had a strong effect on improving the quality of life of patients with first-episode schizophrenia (SMD = −1.26, *p* < 0.001, 95% CI = −1.74 to −0.79, *I*^2^ = 55.0%, k = 2, *n* = 188, Supplementary Figure 9) (38, 39). The clubhouse model of psychiatric rehabilitation had a strong effect on improving the quality of life of patients with schizophrenia when it was performed for 3 months (SMD = −1.01, *p* ≤ 0.001, 95% CI = −1.31 to −0.70, *I*^2^ = 0.0%, k = 2, *n* = 188, Supplementary Figure 10) (38, 39) and 6 months (SMD = −0.91, *p* = 0.01, 95% CI = −1.64 to −0.19, *I*^2^ = 85%, k = 3, *n* = 239, Supplementary Figure 10) (36, 38, 39).

#### Depressive and Anxiety Symptoms

The clubhouse model of psychiatric rehabilitation had a strong effect on promoting the remission of depressive (SMD = −2.33, *p* ≤ 0.001, 95% CI = −2.71 to −1.96, *I*^2^ = 0%, k = 2, *n* = 201, Figure 3) (37, 41) and anxiety symptoms (SMD = −2.03, *p* ≤ 0.001, 95% CI = −2.92 to −1.14, *I*^2^ = 84%, k = 2, *n* = 201, Figure 3) (37, 41) of patients with schizophrenia.

#### Employment and Relapse

Only one randomized controlled study (36) reported the employment and relapse data. Seven participants had a transitional employment in the intervention group (7/28), and one participant had a transitional employment in the control group (1/23). Three participants were re-hospitalized in the intervention group (3/28), and seven participants were re-hospitalized in the control group (7/23).

### Publication Bias

The funnel plots of psychiatric symptoms and social functioning are exhibited in Supplementary Figures 11, 12, respectively. No obvious publication bias was detected. Due to the limited data, the funnel plots of other domains were not exhibited.

### Non-randomized Controlled Trials

Four longitudinal, case-controlled studies (29, 32, 34, 35) reported the treatment effectiveness of the clubhouse model of psychiatric rehabilitation on psychiatric symptoms. Two studies (29, 35) reported that engaging in the clubhouse could promote the remission of psychiatric symptoms. Two other studies (32, 34) did not reach the same conclusion. Two studies (29, 32) reported that the clubhouse model of psychiatric rehabilitation resulted in more effective recovery of negative symptoms than medication treatment alone. One study (35) reported that the clubhouse model of psychiatric rehabilitation resulted in more effective recovery of positive and negative symptoms than medication treatment alone. Three studies (30–32) found that the clubhouse model of psychiatric rehabilitation could promote recovery in functioning of patients with schizophrenia. Four studies (30, 31, 33, 34) demonstrated significant improvements in the quality of life of participants. One study (32) found that family burden was significantly reduced after the intervention. The clubhouse model of psychiatric rehabilitation had a significant treatment effect on depression symptoms of patients with schizophrenia (29). Two studies (29, 30) reported that the clubhouse model of psychiatric rehabilitation could improve the self-esteem of participants.

## Discussion

To the best of our knowledge, the present article is the first meta-analysis about the treatment effect of the clubhouse model of psychiatric rehabilitation on Chinese with schizophrenia. Seven randomized controlled studies with 682 participants (286 males and 396 females) were included in the present meta-analysis. According to the pooled data, the clubhouse model of psychiatric rehabilitation had a significant effect on promoting the remission of psychiatric symptoms, social functioning recovery, reducing the family burden, improving the quality of life, and promoting the remission of depressive and anxiety symptoms. Although data about employment and relapse were limited, the clubhouse model of psychiatric rehabilitation showed a positive effect on these variables.

According to the pooled SMD, the clubhouse model of psychiatric rehabilitation has a strong effect on promoting the remission of psychiatric symptoms. This finding is consistent with the results of some quasi-experimental studies (29, 35). However, some quasi-experimental studies found that the clubhouse model of psychiatric rehabilitation did not have a significant effect on promoting the remission of psychiatric symptoms (32, 34). According to the pooled SMD, engaging in the clubhouse did not have a significant effect on promoting the remission of positive symptoms, but it had a significant effect on promoting the remission of negative symptoms. Two quasi-experimental studies (29, 32) found that engaging in the clubhouse could improve the remission of negative symptoms than positive symptoms. A previous meta-analysis about social skills training for patients with schizophrenia obtained similar results: social skills training had moderate mean effect sizes for negative symptoms and small mean effect sizes for other symptoms (43). The following reasons may explain no benefit to positive symptoms by engaging in the clubhouse: (1) Participants were in a stable stage (they had low positive symptom scores) and underwent medicine treatment while engaging in the clubhouse. (2) Limited studies and participants were included, and there was high heterogeneity across studies. Given that medication has a more effective effect in controlling positive symptoms than negative symptoms, the participants all have higher scores in negative symptoms than positive symptoms at baseline, indicating that the participants had few positive symptoms to be improved. Thus, more randomized controlled studies including participants who have severe positive symptoms are warranted to ensure the effectiveness of the intervention in improving positive symptoms. There was substantial heterogeneity across studies, which may come from the different scales applied to assess psychiatric symptom severity, the participants, or the time of intervention. When we pooled the outcomes that were assessed with PANSS or came from participants with first-episode schizophrenia, the results did not change. But the heterogeneity across studies was still high. When synthesizing the outcomes assessed at 3, 6, and 12 months, we found that 12 months of intervention had a stronger effect on promoting the remission of psychiatric symptoms than 6 months of intervention, and 6 months of intervention had a stronger effect than 3 months of intervention. The heterogeneity was low in synthesizing the outcomes assessed at 3 and 6 months, but it was high in synthesizing the outcomes assessed at 12 months. These findings indicated that at least in the first year engaging in clubhouse, the longer time engaged might get a better outcome in promoting psychiatric symptoms remission.

The clubhouse model of psychiatric rehabilitation had a strong effect on promoting social functioning recovery. Some quasi-experimental studies conducted in China (30–32, 43) and other countries (20, 44) reported the same results. When synthesizing the outcomes assessed at 3 and 6 months, the results did not change. The clubhouse model of psychiatric rehabilitation also had a strong effect on promoting social functioning recovery of patients with first-episode schizophrenia. Clubhouses provide social events, work experiences, and housing to strengthen and increase the social networks of people with SMI. McKay et al. believed that clubhouses provide a useful vehicle for increasing social competence and social integration and promoting recovery (19).

The clubhouse model of psychiatric rehabilitation had a significant effect on reducing the family burden. A quasi-experimental study conducted in China reported the same results (32). We pooled the data of patients with first-episode schizophrenia, and the results did not change. We found that the effect size of reducing the family burden was medium and large when engaging in the clubhouse for 3 and 6 months, respectively. This finding indicated that engaging in the clubhouse for more than 6 months might be more possible to reduce the family burden. In 2013, the economic burden of mental disorders in China was estimated to be 1.1% of the gross domestic product (45). China is characterized by Confucian social ethics, which means that Chinese rely more on the family and organized civil society. The family is regarded as the main caregivers for people with mental disorders. According to our results, the clubhouse model of psychiatric rehabilitation shines some lights on reducing the family burden of patients with schizophrenia.

The clubhouse model of psychiatric rehabilitation had a significant effect on improving the quality of life of patients with schizophrenia. We synthesized the data of patients with first-episode schizophrenia, and the data assessed when patients engaged in the clubhouse for 3 or 6 months, the results did not change. Some quasi-experimental studies conducted in China (30, 31, 33, 34) and other countries (21, 44, 46) reported the same results. However, which factor is associated with the quality of life in schizophrenia remains unclear. Narvaez et al. found that the severity of depressive and negative symptoms and neuropsychological functioning were related to the quality of life of patients with schizophrenia (47). A meta-analysis found that except for negative symptoms, the severity of positive symptoms was related to the quality of life of patients with schizophrenia (48). Many sociodemographic and clinical variables such as gender, marital status, income, and type and amount of medication are also related to the quality of life of patients with schizophrenia (49). We speculate that clubhouse members attain higher quality of life by promoting the recovery of several domains such as negative symptom remission, social functioning recovery, reduction of family burden, remission of depressive and anxiety symptoms, and being employed.

The clubhouse model of psychiatric rehabilitation had a strong effect on promoting the remission of depressive and anxiety symptoms of patients with schizophrenia. However, the conclusion may not be reliable because of the limited number of studies included. A quasi-experimental study conducted in China found that clubhouse members did not achieve better recovery in depressive symptoms compared with non-members of the clubhouse (29). In the present meta-analysis, one randomized controlled study and one quasi-experimental study reported results about employment and relapse (29). The clubhouse model of psychiatric rehabilitation may contribute to increasing the employment rate and reducing the relapse rate. Two reviews concluded that a moderate level of evidence existed regarding the effectiveness of clubhouses in increasing the rate of employment and reducing the rate of relapse of members (19, 50).

In the present meta-analysis, we found that the clubhouse model of psychiatric rehabilitation could promote the remission of symptoms and functional recovery of patients with schizophrenia. A greater flexibility to interpret and reinterpret the clubhouse model is needed to replicate the meaningful clubhouse model in other cultures (23). Although clubhouses are supposed to follow the International Clubhouse Standards, they need to make adaptations according to national and local contexts (24). The clubhouse model of psychiatric rehabilitation may be suitable to address the urgent need for better mental health services in China. However, several concerns need to be addressed. In particular, the clubhouse model of psychiatric rehabilitation might only benefit patients with a stable condition and would have limited applicability (15). Because in China once people with mental disorders access a stable stage, most of them do not want to take part in unpaid psychiatric rehabilitation programs. Instead, they choose to earn money even through a menial job to support their families' finances (15). Moreover, some other concerns about the high cost of certification and training and the need for support from the local government and close multidisciplinary cooperation were raised. The successful and widespread application of the clubhouse model of psychiatric rehabilitation requires a comprehensive consideration of the culture, economy, and politics of China. Accessing support from the local government in politics and finance, addressing mental health workforce development and educational training issues, forming a close multidisciplinary teamwork, and adherence to the core principles and localization of the services are essential for the successful application of the clubhouse model of psychiatric rehabilitation.

## Limitations

This study has some limitations. First, the pooled results should be interpreted with caution because all statistical tests (including *I*^2^ applied in the present study) may have low statistical power due to the small number of included studies and the CIs of *I*^2^ can be large (51). Second, the small number of studies included and the substantial heterogeneity across studies diminish the reliability of the results. Third, the SMD is a statistical index, which cannot represent the true level of recovery. Fourth, the effect of the clubhouse model of psychiatric rehabilitation on some domains of personal recovery such as self-esteem and personal meaning remains unclear because only two quasi-experimental studies reported that the clubhouse model of psychiatric rehabilitation could improve the self-esteem of participants as we mentioned above and none of the included studies reported the effect of the clubhouse model of psychiatric rehabilitation on personal meaning. Fifth, as fidelity to clubhouse standards was not reported, fidelity of the studied clubhouses cannot be confirmed. More high-quality randomized controlled studies about the treatment effects of the clubhouse model of psychiatric rehabilitation are needed.

## Conclusions

The clubhouse model of psychiatric rehabilitation has a significant effect on promoting the remission of psychiatric symptoms, social functioning recovery, reducing the family burden, improving the quality of life, and promoting the remission of depressive and anxiety symptoms of Chinese with schizophrenia. This model may be suitable to address the urgent need for better mental health services in China. Aside from following the International Clubhouse Standards, clubhouses need to make adaptations according to national and local contexts.

## Data Availability Statement

The raw data supporting the conclusions of this article will be made available by the authors, without undue reservation.

## Author Contributions

HY designed the study and created the first draft of the manuscript. WG, YD, and HY conducted the literature search, study selection, quality assessment, and statistical analysis. WG and YD made improvements of the manuscript. All of the authors contributed to the final work.

## Funding

This study was supported by grants from the National Natural Science Foundation of China (Grant No. 81771447).

## Conflict of Interest

The authors declare that the research was conducted in the absence of any commercial or financial relationships that could be construed as a potential conflict of interest.

## Publisher's Note

All claims expressed in this article are solely those of the authors and do not necessarily represent those of their affiliated organizations, or those of the publisher, the editors and the reviewers. Any product that may be evaluated in this article, or claim that may be made by its manufacturer, is not guaranteed or endorsed by the publisher.
