# Supplementary material for: Clubhouse Model of Psychiatric Rehabilitation in China to Promote Recovery of People With Schizophrenia: A Systematic Review and Meta-Analysis
Source: Front Psychiatry. 2021 Sep 13;12:730552. doi: 10.3389/fpsyt.2021.730552 (PMC8473690; doi:10.3389/fpsyt.2021.730552)
Supplement: Supplementary file 1 [file Image_1.PDF]

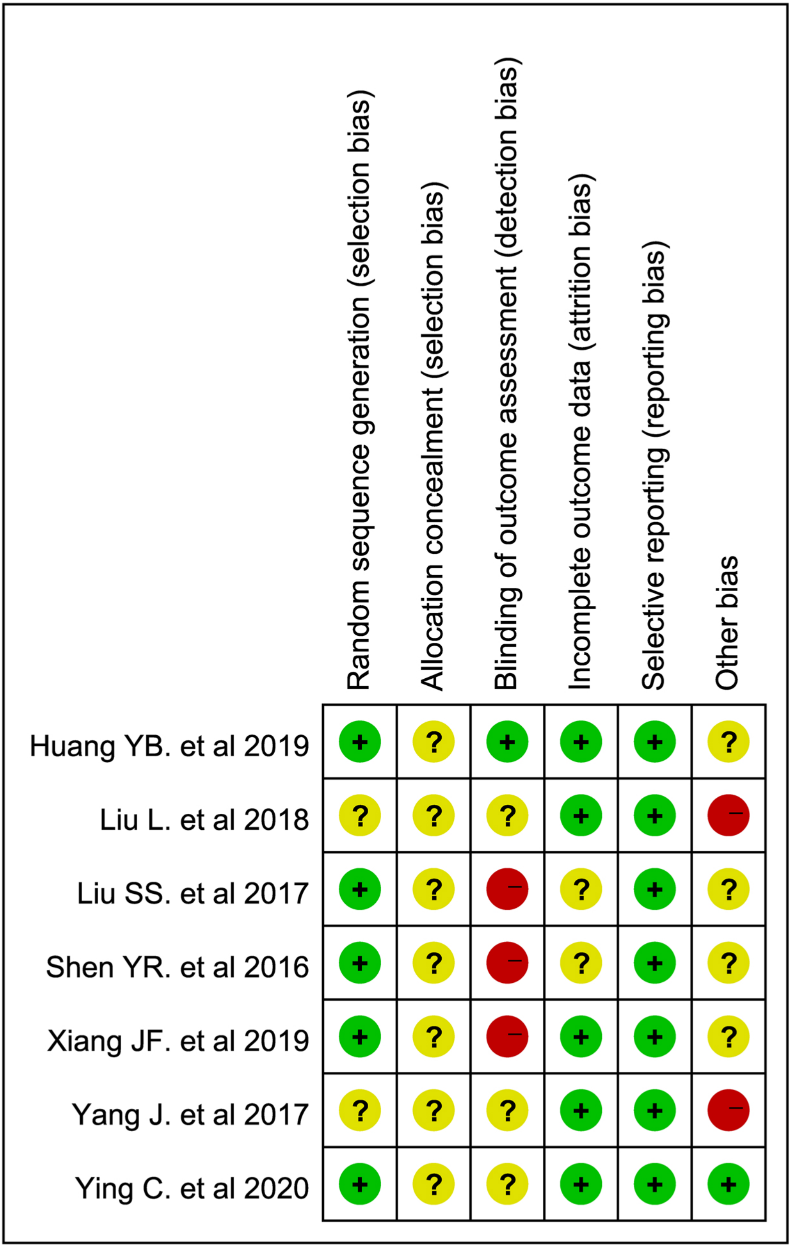
Random sequence generation (selection bias)
Allocation concealment (selection bias)
Blinding of outcome assessment (detection bias)
Incomplete outcome data (attrition bias)
Selective reporting (reporting bias)
Other bias
Huang YB. et al 2019
Liu L. et al 2018
Liu SS. et al 2017
Shen YR. et al 2016
Xiang JF. et al 2019
Yang J. et al 2017
Ying C. et al 2020
