# Supplementary figures and images for: Clubhouse Model of Psychiatric Rehabilitation in China to Promote Recovery of People With Schizophrenia: A Systematic Review and Meta-Analysis
Source: Front Psychiatry. 2021 Sep 13;12:730552. doi: 10.3389/fpsyt.2021.730552 (PMC8473690; doi:10.3389/fpsyt.2021.730552)

**Figure S2: Pooled SMD about psychiatric symptoms which assessed with different scales**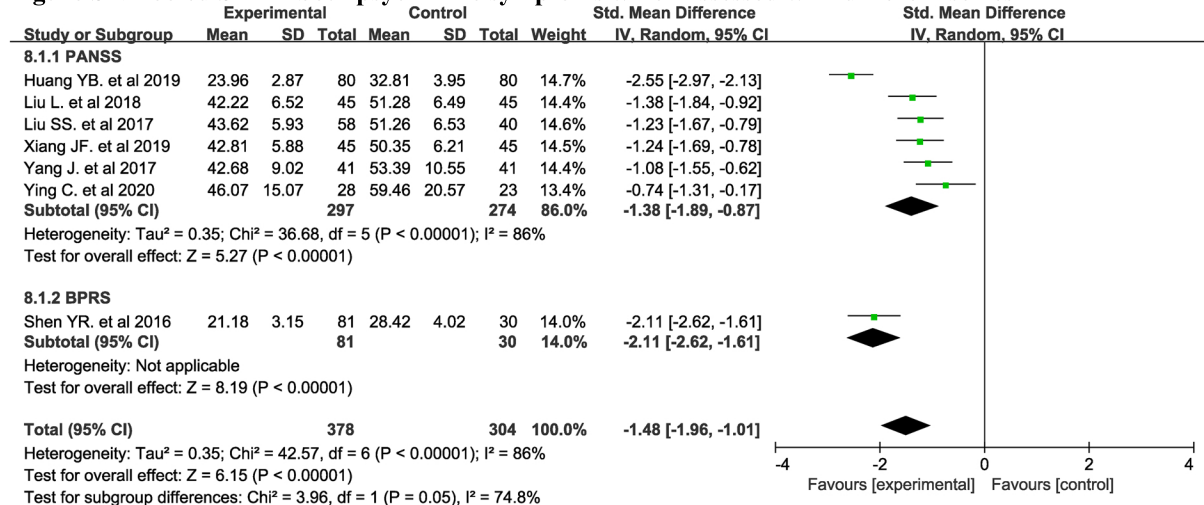

Supplement: Supplementary file 2 [file Image_2.PDF]

**Figure S5: Pooled SMD about social functioning for patients with first-episode schizophrenia**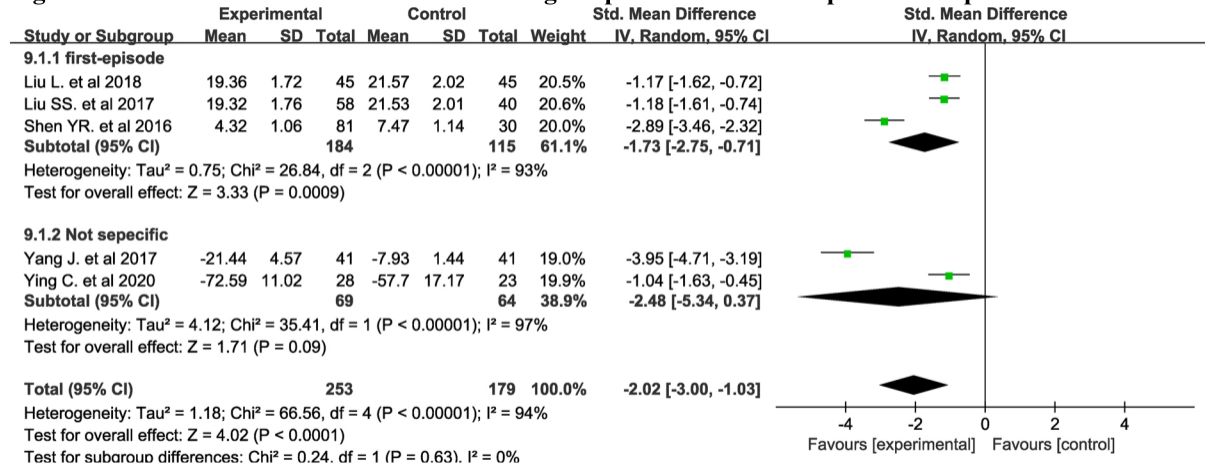

Supplement: Supplementary file 5 [file Image_5.PDF]

**Figure S6: Pooled SMD about social functioning which assessed at different time point**

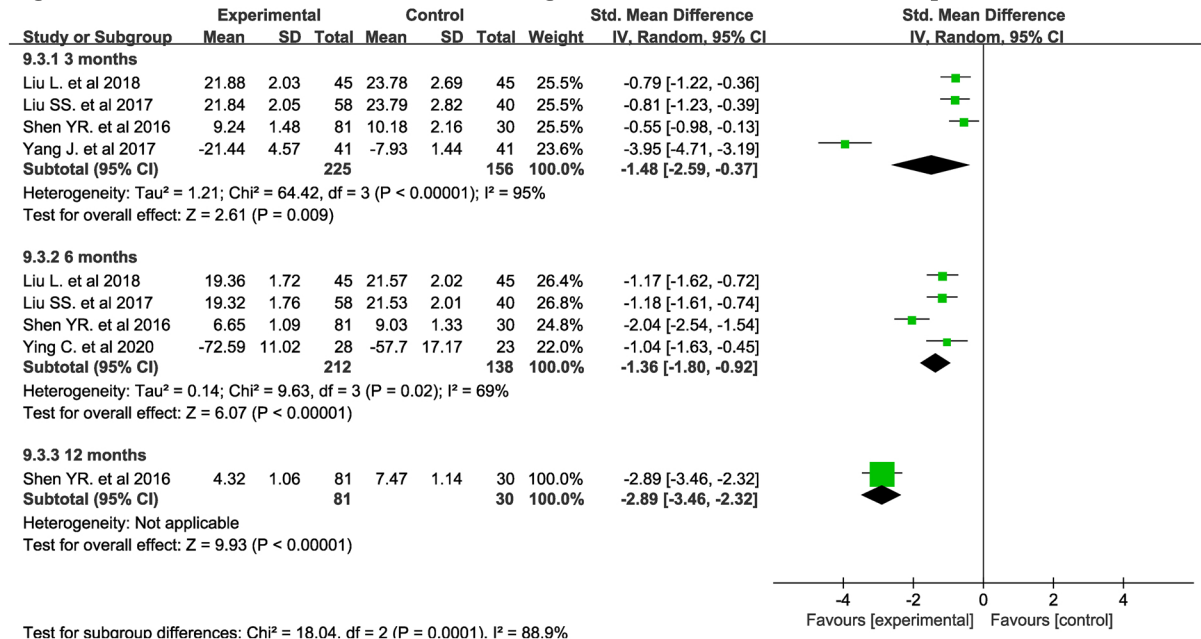

Supplement: Supplementary file 6 [file Image_6.PDF]

**Figure S7: Pooled SMD about family burden for patients with first-episode schizophrenia**

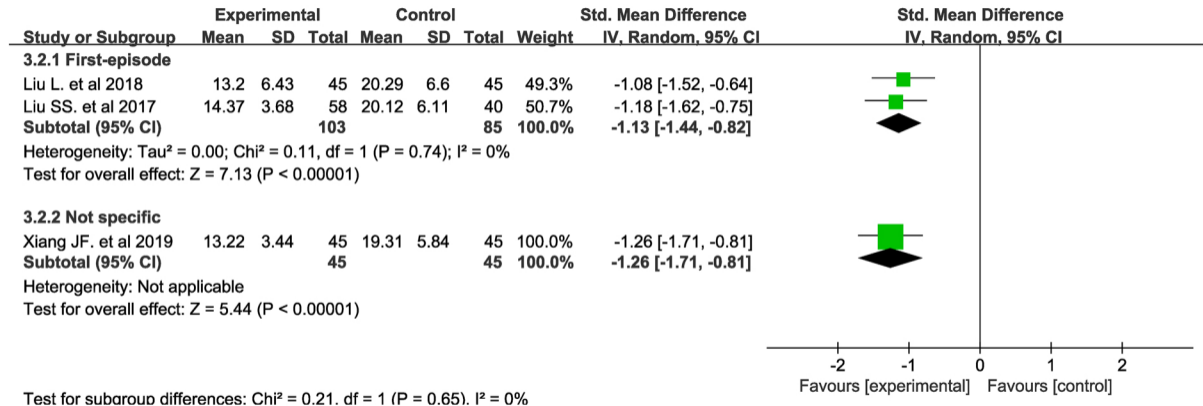

Supplement: Supplementary file 7 [file Image_7.PDF]

**Figure S8: Pooled SMD about family burden which assessed at different time point**

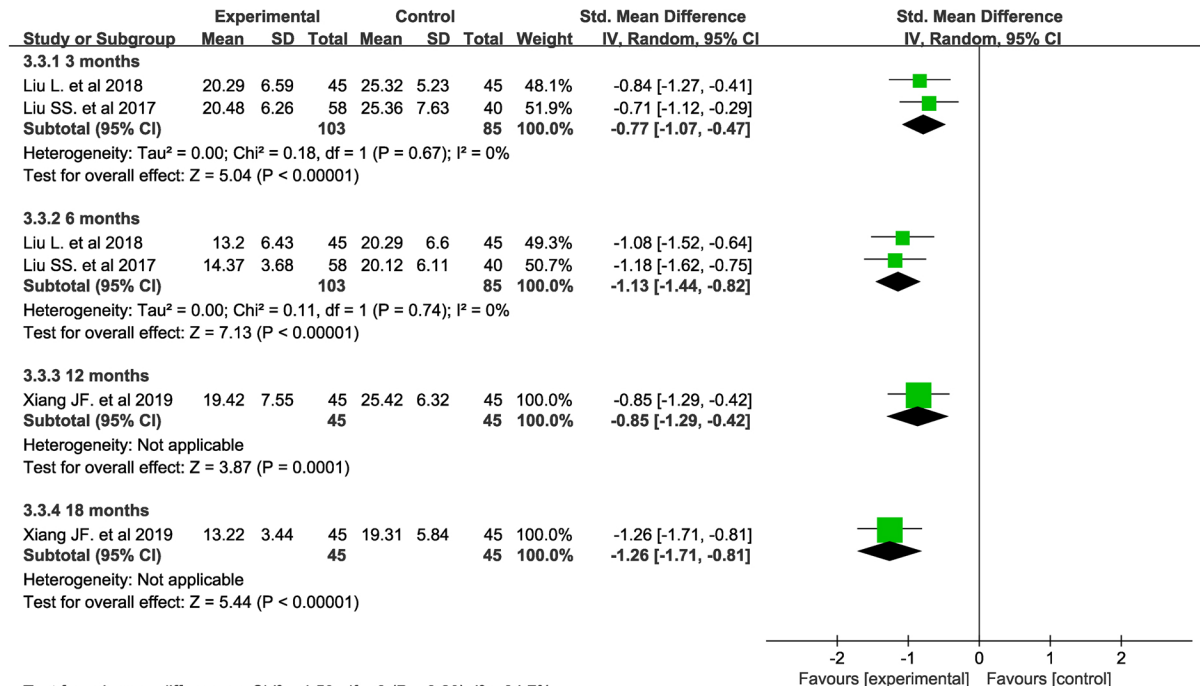

Supplement: Supplementary file 8 [file Image_8.PDF]

**Figure S9: Pooled SMD about quality of life for patients with first-episode schizophrenia**

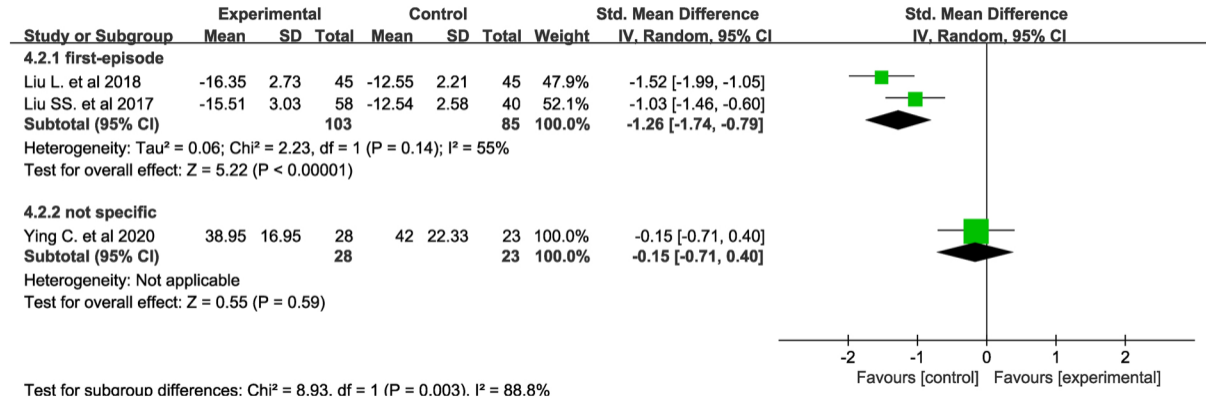

Supplement: Supplementary file 9 [file Image_9.PDF]

**Figure S10: Pooled SMD about quality of life which assessed at different time point**

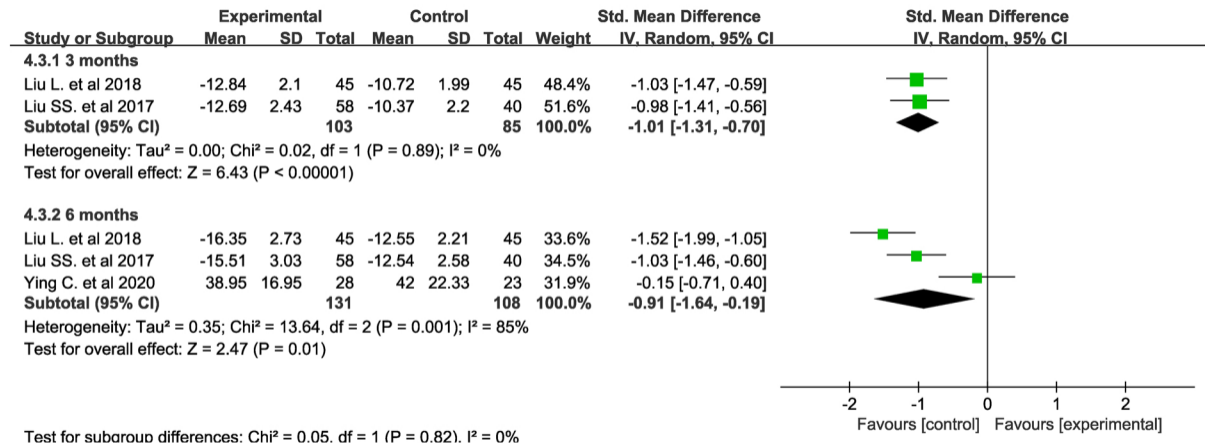

Supplement: Supplementary file 10 [file Image_10.PDF]

**Figure S11: Funnel plot of psychiatric symptoms**

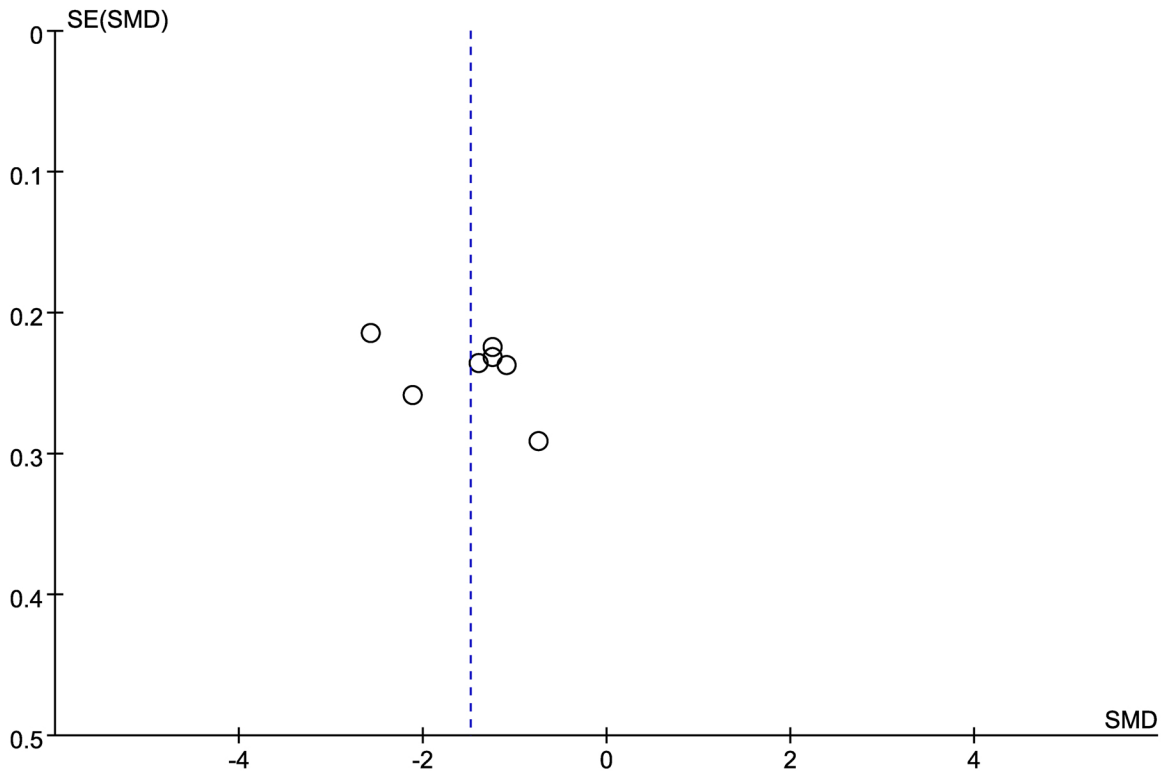

Supplement: Supplementary file 11 [file Image_11.PDF]

**Figure S12: Funnel plot of social functioning**

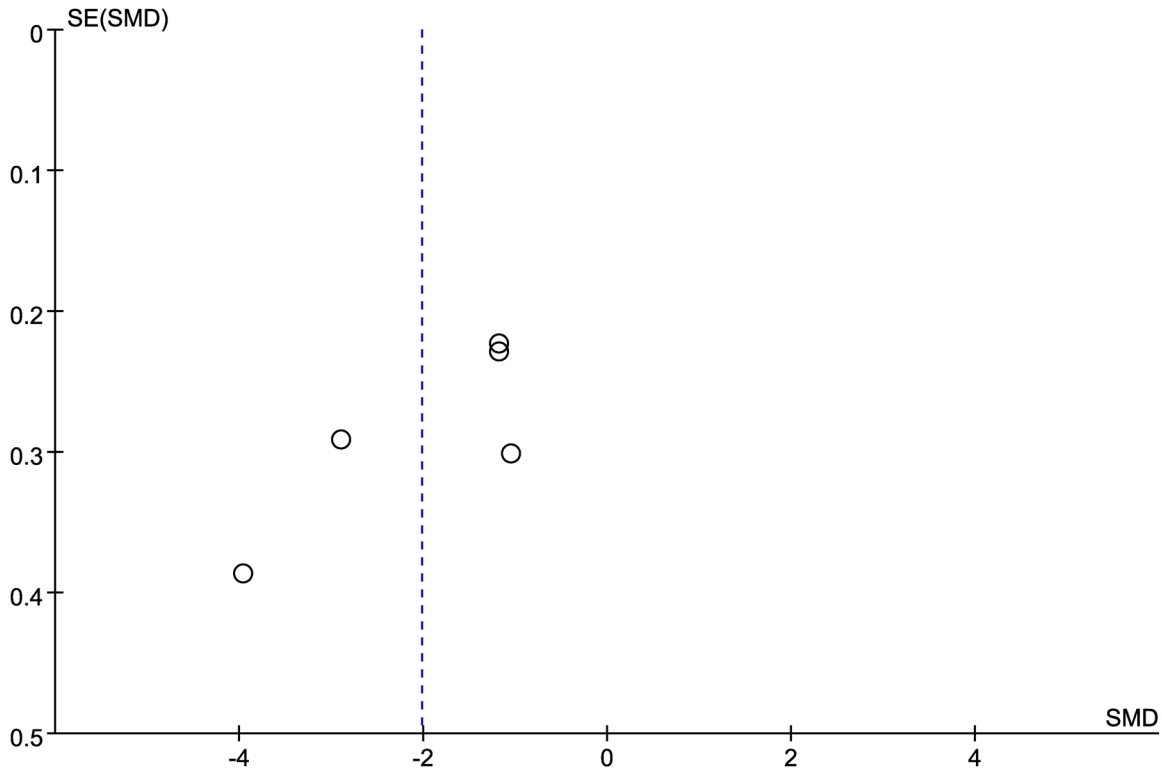

Supplement: Supplementary file 12 [file Image_12.PDF]
