# Supplementary material for: Clubhouse Model of Psychiatric Rehabilitation in China to Promote Recovery of People With Schizophrenia: A Systematic Review and Meta-Analysis
Source: Front Psychiatry. 2021 Sep 13;12:730552. doi: 10.3389/fpsyt.2021.730552 (PMC8473690; doi:10.3389/fpsyt.2021.730552)

Figure S3: Pooled SMD about psychiatric symptoms for patients with first-episode schizophrenia

	Experimental			Control				Std. Mean Difference	Std. Mean Difference
Study or Subgroup	Mean	SD	Total	Mean	SD	Total	Weight	IV, Random, 95% CI	IV, Random, 95% CI
Huang YB. et al 2019	23.96	2.87	80	32.81	3.95	80	25.5%	-2.55 [-2.97, -2.13]	
Liu L. et al 2018	42.22	6.52	45	51.28	6.49	45	25.1%	-1.38 [-1.84, -0.92]	
Liu SS. et al 2017	43.62	5.93	58	51.26	6.53	40	25.4%	-1.23 [-1.67, -0.79]	
Ying C. et al 2020	46.07	15.07	28	59.46	20.57	23	24.0%	-0.74 [-1.31, -0.17]	
**Total (95% CI)**			**211**			**188**	**100.0%**	**-1.49 [-2.25, -0.72]**	

Heterogeneity: $Tau^2 = 0.55$; $Chi^2 = 32.01$, df = 3 ($P < 0.00001$); $I^2 = 91\%$

Test for overall effect: Z = 3.82 (P = 0.0001)

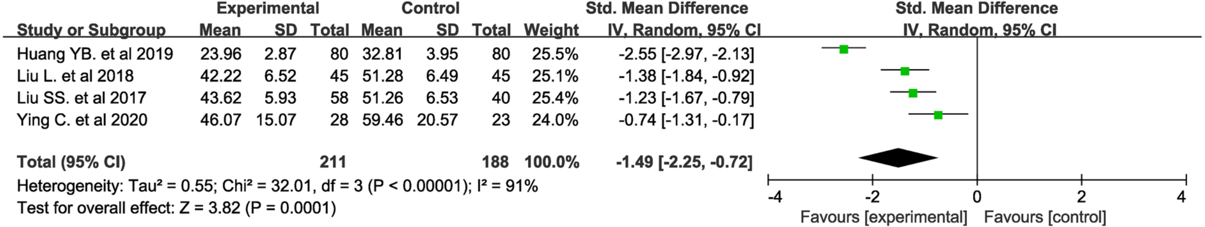

Supplement: Supplementary file 3 [file Image_3.PDF]
