# Supplementary material for: Clubhouse Model of Psychiatric Rehabilitation in China to Promote Recovery of People With Schizophrenia: A Systematic Review and Meta-Analysis
Source: Front Psychiatry. 2021 Sep 13;12:730552. doi: 10.3389/fpsyt.2021.730552 (PMC8473690; doi:10.3389/fpsyt.2021.730552)
Supplement: Supplementary file 4 [file Image_4.PDF]

Figure S4: Pooled SMD about psychiatric symptoms which assessed at different time point

	Experimental			Control				Std. Mean Difference
Study or Subgroup	Mean	SD	Total	Mean	SD	Total	Weight	IV, Random, 95% CI
**8.3.1 3 months**								
Liu L. et al 2018	50.29	7.8	45	55.09	7.53	45	25.5%	-0.62 [-1.04, -0.20]
Liu SS. et al 2017	50.33	7.4	58	55.08	7.76	40	26.4%	-0.62 [-1.04, -0.21]
Shen YR. et al 2016	30.26	4.12	81	32.01	4.38	30	25.6%	-0.41 [-0.84, 0.01]
Yang J. et al 2017	42.68	9.02	41	53.39	10.55	41	22.5%	-1.08 [-1.55, -0.62]
**Subtotal (95% CI)**			**225**			**156**	**100.0%**	**-0.67 [-0.94, -0.41]**
Heterogeneity: Tau² = 0.02; Chi² = 4.50, df = 3 (P = 0.21); I² = 33%								
Test for overall effect: Z = 5.00 (P < 0.00001)								
**8.3.2 6 months**								
Liu L. et al 2018	42.22	6.52	45	51.28	6.49	45	26.2%	-1.38 [-1.84, -0.92]
Liu SS. et al 2017	43.62	5.93	58	51.26	6.53	40	28.9%	-1.23 [-1.67, -0.79]
Shen YR. et al 2016	25.56	3.84	81	30.38	4.18	30	27.7%	-1.22 [-1.67, -0.77]
Ying C. et al 2020	46.07	15.07	28	59.46	20.57	23	17.2%	-0.74 [-1.31, -0.17]
**Subtotal (95% CI)**			**212**			**138**	**100.0%**	**-1.18 [-1.42, -0.94]**
Heterogeneity: Tau² = 0.00; Chi² = 3.04, df = 3 (P = 0.38); I² = 1%								
Test for overall effect: Z = 9.71 (P < 0.00001)								
**8.3.3 12 months**								
Huang YB. et al 2019	23.96	2.87	80	32.81	3.95	80	33.5%	-2.55 [-2.97, -2.13]
Shen YR. et al 2016	21.18	3.15	81	28.42	4.02	30	33.0%	-2.11 [-2.62, -1.61]
Xiang JF. et al 2019	50.67	7.2	45	54.02	6.98	45	33.5%	-0.47 [-0.89, -0.05]
**Subtotal (95% CI)**			**206**			**155**	**100.0%**	**-1.71 [-3.02, -0.40]**
Heterogeneity: Tau² = 1.28; Chi² = 51.46, df = 2 (P < 0.00001); I² = 96%								
Test for overall effect: Z = 2.56 (P = 0.01)								
**8.3.4 18 months**								
Xiang JF. et al 2019	42.81	5.88	45	50.35	6.21	45	100.0%	-1.24 [-1.69, -0.78]
**Subtotal (95% CI)**			**45**			**45**	**100.0%**	**-1.24 [-1.69, -0.78]**
Heterogeneity: Not applicable								
Test for overall effect: Z = 5.35 (P < 0.00001)								

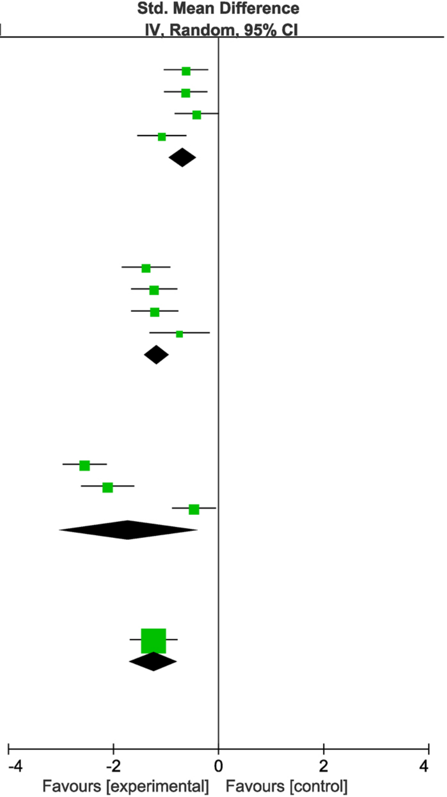

Test for subgroup differences: Chi² = 10.33, df = 3 (P = 0.02), I² = 71.0%
