# Supplementary material for: Clubhouse Model of Psychiatric Rehabilitation in China to Promote Recovery of People With Schizophrenia: A Systematic Review and Meta-Analysis
Source: Front Psychiatry. 2021 Sep 13;12:730552. doi: 10.3389/fpsyt.2021.730552 (PMC8473690; doi:10.3389/fpsyt.2021.730552)
Supplement: Supplementary file 13 [file Table_1.DOCX]

Table S1. Characteristics of the included non-randomized controlled studies.

| Author, year | City | Participants | Diagnosis | Intervention | The time of intervention | Experimental group: sample size and demographic characteristic | Control group: sample size and demographic characteristic | Dropout data |
| --- | --- | --- | --- | --- | --- | --- | --- | --- |
| Yang J. et al, 2017 | Chongqin | Patients with schizophrenia | ICD‐10 | Experimental group: A;  Control group: None | 3 months | 45 (Man: 23; Age: 39±10 (y); Illness duration: 1-120 months; Educational level: Primary 4; Secondary 26; Tertiary 15) | 45 (Man: 20; Age 40±10 (y); Illness duration: 1-120 months; Educational level: Primary 5, Secondary 28, Tertiary 12) | clubhouse 3; control 3 |
| Wu HJ. et al, 2019 | Hangzhou | Patients with schizophrenia | CCMD‐3 | Experimental group: A;  Control group: B | 12 months | 67 (Man: 40; Age: 41.61±8.20 (y); Marital status: Single 48, Married 4, Separated 15) | 71 (Man: 37; Age: 41.61±10.24 (y); Marital status: Single 49, Married 12, Separated 10) | clubhouse 0; control 0 |
| Shen YR. et al, 2013 | Shenzhen | Patients with schizophrenia | CCMD‐3 | Experimental group: A;  Control group: B | 12 months | 17 (Man: 11; Illness duration 15.88±6.57 (y); Age: 39.23±8.71 (y)) | 76 (Man: 38; Illness duration 17.17±8.42 (y); Age: 41.82±10.39) | clubhouse 0; control 18 |
| Huang GH. et al, 2016 | Enshi | Patients with schizophrenia | CCMD‐3 | Experimental group: A;  Control group: B | 6 months | 50 | 50 | NA |
| Hong L. et al, 2015 | Chengdu | Patients with schizophrenia | ICD‐10 | Experimental group: A;  Control group: None | 3 months | 26 | 36 | clubhouse 10; control 20 |
| He J. et al, 2012 | Changsha | Patients with schizophrenia | ICD‐10 or CCMD‐3 | Experimental group: A;  Control group: None | 24 months | 81 (Man: 45; Age: 37.43±8.2 (y); Illness duration:6±3.1 (y)) | 66 (Man: 37; Age: 39.25±9.1 (y); Illness duration: 7±4.3 (y)) | clubhouse 2; control 6 |
| Tsang. et al, 2010 | Hong Kong | Patients with schizophrenia | ICD‐10 | Experimental group: A;  Control group: None | 6 months | 46 (Man: 31; Age: 40.5 ± 8.8 (y); Illness duration: 17.4 ± 9.8 (y); Educational level: Primary 5, Secondary 37, Tertiary 4; Marital status: Single 34, Married 9, Separated 3) | 46 (Man: 31; Age: 40.3 ± 8.8 (y); Illness duration: 15.7 ± 9.3 (y); Educational level: Primary 8, Secondary 33, Tertiary 5; Marital status: Single 36, Married 5, Separated 5) | clubhouse 7; control 5 |

CCMD‐3: the third version of Chinese Classification of Mental Disorders; ICD‐10: the 10th version of the International Statistical Classification of Diseases and Related Health Problems; A: clubhouse model of psychiatric rehabilitation; B: general psychiatric rehabilitation; NA: not available.
