# Supplementary material for: Clubhouse Model of Psychiatric Rehabilitation in China to Promote Recovery of People With Schizophrenia: A Systematic Review and Meta-Analysis
Source: Front Psychiatry. 2021 Sep 13;12:730552. doi: 10.3389/fpsyt.2021.730552 (PMC8473690; doi:10.3389/fpsyt.2021.730552)
Supplement: Supplementary file 14 [file Table_2.DOCX]

Table S2. Quality assessment of non-randomized studies using the Mixed Methods Appraisal Tool.

| Quasi-experimental  studies | 1, Are the participants representative of the target population? | 2, Are measurement appropriate regarding both the outcome and intervention (or exposure)? | 3, Are there Complete outcome data? | 4, Are the confounders accounted for in the design and analysis? | 5, During the study period, is the intervention administered (or exposure occurred) as intended? |
| --- | --- | --- | --- | --- | --- |
| Yang J. et al, 2017 | Yes | Yes | Yes | No | Yes |
| Wu HJ. et al, 2019 | Yes | Yes | Yes | No | Yes |
| Shen YR. et al, 2013 | Yes | Yes | Yes | No | Yes |
| Huang GH. et al, 2016 | Yes | Yes | Cannot tell | Yes | Yes |
| Hong L. et al, 2015 | Yes | Yes | Yes | No | Yes |
| He J. et al, 2012 | Yes | Yes | Yes | No | Yes |
| Tsang. et al, 2010 | Yes | Yes | Yes | Yes | Yes |
